# Successful Management of Intravascular Hemolysis Caused by Systemic Loxoscelism With Antivenom: A Case Report

**DOI:** 10.7759/cureus.91678

**Published:** 2025-09-05

**Authors:** Claudia Guadarrama-Fernández, José A Longino, Norma Villalba-Ríos, Mario A Rojas-Alanís, Carlos R Cervantes-Sánchez

**Affiliations:** 1 Department of Emergency Services, Hospital General de Chihuahua “Dr. Salvador Zubirán Anchondo”, Chihuahua, MEX; 2 Intensive Care Unit, Hospital General de Chihuahua “Dr. Salvador Zubirán Anchondo", Chihuahua, MEX; 3 Department of Toxicology, Pensiones Civiles del Estado de Chihuahua, Chihuahua, MEX; 4 Department of Educational Research and Innovation, Facultad de Medicina y Ciencias Biomédicas, Universidad Autónoma de Chihuahua, Chihuahua, MEX; 5 Department of Surgery, Hospital General de Chihuahua "Dr. Salvador Zubirán Anchondo", Chihuahua, MEX

**Keywords:** antivenom, envenomation, fabotherapy, hemolytic anemia, loxoscelism

## Abstract

Spider bites, particularly from *Loxosceles* species, pose a public health concern in Mexico. A case of systemic loxoscelism in a 33-year-old woman who developed acute intravascular hemolysis is presented. The patient was successfully treated with blood transfusions and Reclusmyn, a Mexican antivenom. Her clinical and laboratory improvement supports the therapeutic value of antivenom in severe envenomation. This case highlights the importance of early suspicion, recognition, and targeted treatment to prevent complications and improve outcomes.

## Introduction

*Loxosceles* spider envenomation is a medical condition resulting from the bite of the commonly referred to as the Brown Recluse spider. This condition can lead to various systemic manifestations, including hematologic complications (such as acute hemolytic anemia, disseminated intravascular coagulation), and acute renal failure. The severity of the envenomation is influenced by factors, among which can be listed the species of the spider, the amount of venom injected, and host factors. It is important for medical providers, especially those who practice in areas where *Loxosceles* spiders are endemic, to be familiar with the potential hematologic and systemic complications associated with the spider's bite envenomation [1, 2].

There are approximately 140 species of *Loxosceles* spiders (World Spider Catalog 2025), and *L. laeta, L. reclusa*, and *L. deserta* are considered to be the most important *Loxosceles* species in the world, due to their wide geographical distribution and the large number of bites reported with considerable morbidity and mortality [3]. Nevertheless, in North America, Mexico is the country with the highest diversity of *Loxosceles* spiders, with 33 species (World Spider Catalog 2025) [3]. According to the Epidemiological Bulletin of the Mexican Secretariat of Health, 11% of the total number of envenomations is caused by spider bites and is considered a public health problem, with 3,000 annual reports of *Loxosceles* spider bites [1, 4]. In the state of Chihuahua in Mexico, the most common species are* L. arizonica* and *L. apachea* [5]. However, loxoscelism in Mexico is not well represented in the medical literature, mainly due to the lack of reliable scientific reporting [4].

Case reports are crucial in increasing awareness and understanding of rare or unusual medical conditions, such as *Loxosceles* spider envenomation. They provide valuable insights into the clinical presentation, diagnostic challenges, and management strategies for such clinical conditions. In the context of *Loxosceles* spider envenomation, case reports contribute to the body of medical literature by documenting the diverse manifestations of the condition, the diagnostic approaches employed, and the outcomes of specific management interventions. Furthermore, case reports serve as educational tools for healthcare providers, helping them recognize and effectively manage *Loxosceles* spider envenomation, especially in areas endemic to these spiders [6].

In this case, it is intended to highlight the key clinical, diagnostic, and therapeutic aspects of an affected individual, such as the circumstances of the envenomation incident and the subsequent diagnostic and management processes.

## Case presentation

**Figure 1:**
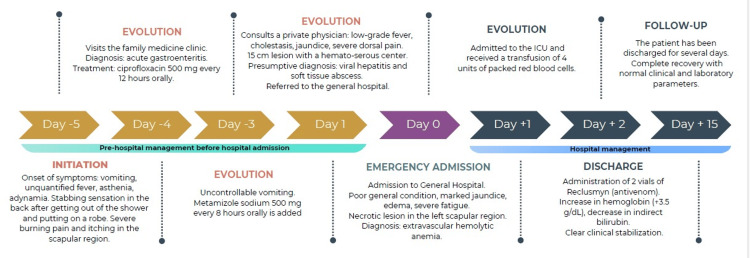
Timeline of clinical events. Clinical timeline diagram prepared by the author.

A 33-year-old female patient presented with symptoms beginning five days before she visited the family medicine unit (Figure 1). Initial signs and symptoms included vomiting of gastric content, unquantified fever, asthenia, and adynamia. She was first suspected of acute gastroenteritis and discharged with ciprofloxacin 500 mg orally every 12 hours. The following day, she returned with uncontrollable vomiting, and metamizole sodium 500 mg orally every eight hours was added to her management.

On the third day, she presented to a private physician with low-grade fever (37.8°C), weakness, fatigue, dry mucous membranes, cholestasis, and generalized jaundice, along with intense pain in the dorsal thoracic region. Physical examination revealed an erythematous cutaneous lesion measuring 15 cm in diameter with a necrotic center. Additionally, she required assistance from family members for proper ambulation. Laboratory findings on referral included elevated transaminases (AST 157 U/L, ALT 196 U/L), total bilirubin 6.54 mg/dL, indirect bilirubin 1.84 mg/dL, and direct bilirubin 4.7 mg/dL. The initial diagnosis was probable viral hepatitis and soft tissue abscess in the posterior thorax, sending the patient to our Hospital, where she was admitted.

**Figure 2:**
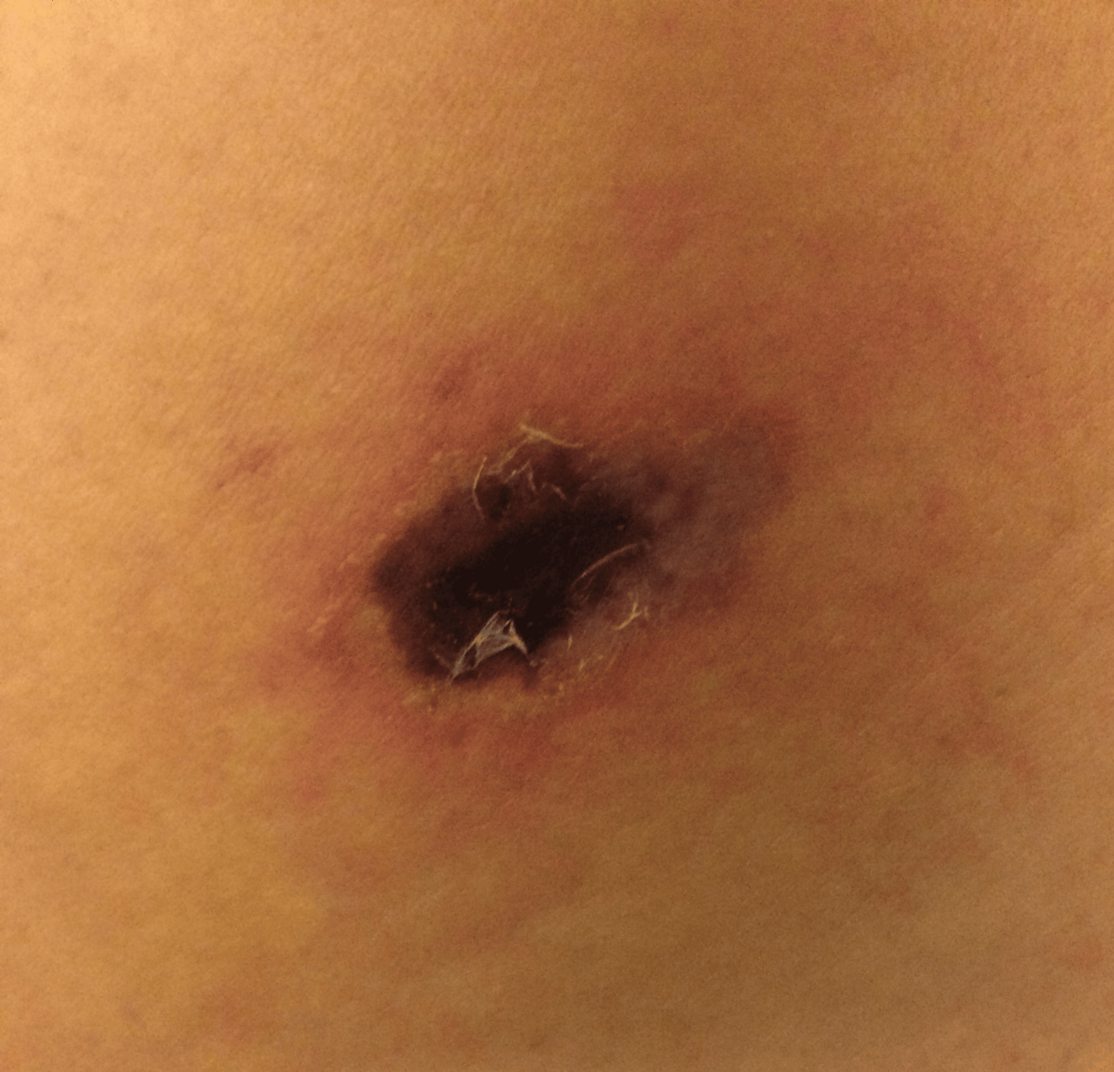
Left para-scapular dermal injury with a large area of peripheral erythema (≈ 6 cm) with a pale halo and another concentric violet halo and necrotic center.

Upon admission to the emergency room at our unit, the patient presented with a five-day clinical picture, demonstrating poor general condition (heart rate (HR) 96 bpm, respiratory rate (RR) 22 x min, blood pressure (BP) 138/84, Temp 37.8°C), marked jaundice, dehydration, and mild edema in the feet and hands, reporting extreme fatigue. After further questioning, the patient reported that the onset of symptoms occurred five days before admission, describing a "stabbing sensation in the back" following a shower and donning a bathrobe, subsequently experiencing intense burning pain with significant pruritus. Clinically, the patient exhibited a lesion on the left scapular region with a large area of peripheral erythema approximately 6 cm in diameter, featuring a pale halo accompanied by a violaceous concentric halo, and a necrotic center, tender to palpation (Figure 2). Generalized jaundice (+++/++++), algic facial expression, adynamia, and poorly hydrated oral mucosa were observed. No hepatomegaly or splenomegaly was palpated upon abdominal examination. An ultrasound of the upper abdomen revealed no hepatic or biliary tract abnormalities. Peripheral blood smear revealed erythroblasts, spherocytes, and erythrocytes in a coin-stack formation.

During her hospital stay, the patient required consultation and transfer to the intensive care unit (ICU) due to extravascular hemolytic anemia (sudden hemoglobin (Hb) fall to less than 7 g/dL). Four packs of red blood cells were transfused, resulting in partial improvement of her hemodynamic status and severe dyspnea, which at one point was considered to require mechanical ventilation. On the second day in the ICU (not intubated, just monitored), she received two vials of Reclusmyn (a fabotherapeutic agent against the venom of the Brown Recluse spider), leading to clinical improvement and a 3.5 g/dL increase in hemoglobin post-packed red blood cell transfusions, without evidence of further hemolysis after that. This was interpreted as a decrease in the hemolytic action of the venom, reflected by a decrease in indirect bilirubin, an increase in hemoglobin, and ongoing clinical improvement, including wound healing, permitting discharge from the hospital with clinical and laboratory parameters within normal ranges three days later.

## Discussion

Only a small percentage of spider species worldwide are of medical importance. The genus *Loxosceles*, known as the violin spider, is responsible for causing dermonecrotic lesions through a unique enzyme, sphingomyelinase D, which is only found in this genus of spiders, besides several bacteria [4]. Envenomation by spiders of the genus *Loxosceles* is called loxoscelism and was first described by Chilean doctors in 1872, known as the “gangrene ulcer of Chile” or “gangrene in flame” [5]. Those spiders have sedentary nocturnal habits, their preferred location being dry, dark places, under rocks or wood, or in the burrows of other animals. In the urban environment, they are located in peri-domiciliary areas and inside homes, hiding behind paintings, furniture, clothes, or shoes [1, 4]. The spider only bites as a last resort defense mechanism when pressed against the human body, as it seems to have been in this case.

Spiders of this genus measure 7-15 mm in length and 0.2-2.5 cm in diameter. The characteristics to identify it are the dark part on the back of the cephalothorax, including an anterior region in the shape of a violin and the "neck" pointing towards the abdomen from which the name "fiddler" comes, as well as the distribution of its six eyes of equal size; two lateral pairs and one central U-shaped pair [1-5].

The venoms of the genus *Loxosceles* are a complex mixture of toxins, especially enriched in three molecular families: phospholipases D, astacin-like metalloproteases, and cystine bond inhibitory peptides [7].

The active toxin is sphingomyelinase D (SMase D), a phospholipase D capable of promoting sphingomyelin hydrolysis to produce ceramide 1-phosphate and lysophosphatidylcholine (LPC), two products that disrupt cell membrane integrity, leading to cell necrosis and the characteristic dermal lesions [1]. However, LPC also produces lysophosphatidic acid, a known inducer of platelet aggregation, endothelial hyperpermeability, and proinflammatory response. Additionally, SMase D directly causes hemolysis in severe cases of systemic envenomation due to complement system activation, leading to the formation of the membrane attack complex, resulting in cell lysis, which can lead to acute hemolytic anemia and even renal impairment [7-9]. Conversely, the activation of leukocytes further exacerbates inflammation, facilitating progressive tissue damage, and neutrophils accumulate and release enzymes that aggravate necrosis [4]. Due to this mechanism, the use of complement activation inhibitors can be considered for future research [7].

The mechanism of intravascular hemolysis secondary to the venom of the genus *Loxosceles* is due to the activation of the alternative complement pathway, due to the activation of metalloproteases from the Adamlysin family by cleaving the sialic acid-rich extracellular domains of glycophorins A, B, and C from erythrocytes [1]. One of the main anticoagulation pathways is that of protein C, which is activated by thrombin, depending on whether it is activated. It binds to thrombomodulin, which increases the ability of thrombin to activate protein C by 1000 times, or to the endothelial protein C receptor, which increases it by 20 times. Disseminated intravascular coagulation is due to the destruction of thrombomodulin and the endothelial protein C receptor by sphingomyelinase D [1, 2, 7-9].

In cases of systemic loxoscelism, the venom’s action can extend beyond the bite site. The toxin may trigger vasculitis, massive hemolysis, and renal dysfunction, likely due to the release of toxic cellular breakdown products and an exaggerated immune response. Severe complications, such as disseminated intravascular coagulation, have been reported in several cases [10].

Clinically, a typical bite manifests a characteristic pattern that includes pruritus, pain, and erythema within six hours, and an irregular erythematous ring delineating the bite at 24 hours. In more severe cases, necrosis may occur within 48-72 hours. The early signs of necrosis are hyperesthesia, bulla, and cyanosis, which involve an ulcer covered by a scab. Ecchymosis may develop centrally to the lesion, followed by a rim of pallor and yet another rim of erythema, which is the “red, white, and blue sign” classic for *L. reclusa* envenomation, but not always present. Anyway, the severity of the injury does not correlate with the development of systemic loxoscelism [10, 11].

Constitutional symptoms include fever, asthenia, nausea, vomiting, erythema, myalgia, arthralgia, seizures, or altered mental status, beginning 48 hours or more after the bite. Systemic loxoscelism occurs most commonly in pediatric, geriatric, and immunocompromised patients. However, severe cases may progress to hemolysis, renal failure, and disseminated intravascular coagulation. Oliguria and hematuria are commonly the first warning signs of severe disease [10].

The number of confirmed cases of loxoscelism is low (23.3%) because the spider is not widely available for identification. A specific immunoassay (ELISA) to detect the presence of venom in the circulation exists as a diagnostic test; however is not available elsewhere. Thus, the diagnosis of loxoscelism remains fundamentally clinical-epidemiological [1].

These tests, in conjunction with the clinical history, indicated that the patient suffered from a process of acute intravascular hemolysis, confirmed by the elevation of indirect bilirubin, jaundice, and a drastic decrease in hemoglobin.

The systemic form of loxoscelism is less common than the cutaneous form, but more serious, including the development of intravascular hemolysis (responsible for hemolytic anemia, jaundice, and hemoglobinuria), intravascular coagulation, and acute renal failure, which is its main cause of death [2].

Management of the dermonecrotic lesion and systemic effects is still controversial, and treatment varies according to severity [1]. Bites can be cleaned and treated with rest, ice, compression, and elevation (RICE). Mild bites can be treated symptomatically with aspirin and antihistamines. Tetanus immunization status must be updated. One should also consider the possibility of an infection secondary to the bite site as the cause of the symptoms. Other strategies may include surgical debridement, skin grafting, hyperbaric oxygen therapy, and vacuum therapy for the treatment of local lesions, as well as antivenom therapy for systemic effects [1, 3, 12, 13].

Antivenom therapy is available in four Latin American countries, including Mexico, which produces Reclusmyn polyspecific antivenom to neutralize the venoms of *L. laeta, L. reclusa*, and* L. boneti*. Most authors consider that its use should be preferred in the treatment of systemic symptoms, as it was chosen in the present case, resulting in a dramatic recovery. However, it is important to consider that this kind of therapy is not innocuous, and several patients could manifest some early anaphylactic reaction [1, 12, 13].

## Conclusions

The administration of Reclusmyn in the present case resulted in significant clinical improvement; after transfusion of packed red blood cells and administration of two vials of antivenom, a sustained increase in hemoglobin and a decrease in indirect bilirubin were observed, with no evidence of subsequent progressive hemolysis. This outcome suggests that the antivenom neutralized the residual effect of the venom, stopping the active hemolytic process and allowing the patient’s hematological and clinical recovery.
